# Supplementary material for: Bridging the gap between in silico and in vivo by modeling opioid disposition in a kidney proximal tubule microphysiological system
Source: Sci Rep. 2021 Nov 1;11:21356. doi: 10.1038/s41598-021-00338-y (PMC8560754; doi:10.1038/s41598-021-00338-y)
Supplement: Supplementary file 1 — Supplementary Information. [file 41598_2021_338_MOESM1_ESM.docx]

**Supplementary Materials Table of Contents**

Materials and Methods

Supplemental Figure 1. Metabolism and transport of morphine using liver MPS

Supplemental Table 1. Donor information on kidney tissues used for isolation of proximal tubule epithelial cells.

Supplemental Table 2. Transport of morphine and M6G by VPT-MPS

Supplemental Table 3. TEER and Papp values for the bidirectional transport of Morphine, M6G and [^3^H]PAH across PTECs and MDCK monolayers after 1.5 hour incubation in conventional 2D Transwell^TM^.

Supplemental Table 4. Prediction of renal clearance of morphine and M6G from PTECs permeability study data

**Supplementary Materials:**

**Materials and Methods.**

### Transport of Morphine and M6G across PTECs monolayers in conventional 2D Transwell^TM^ and prediction of renal clearance from 2D Transwell^TM^ study

PTECs were seeded on polyester membrane HTS 24-well Transwell^TM^ inserts at 0.5-1 ×10^5^ cells/well and cultured for 7-10 days until confluency. Transepithelial electrical resistance

(TEER) across PTECs monolayers was monitored by an EVOM meter from World Precision Instruments (Berlin, Germany). TEER values were normalized to culture surface areas of Transwell^TM^ inserts. Wells with TEER reading from 25-150 ohm∙cm^2^ were used for experiments.

Permeability experiments were carried out by addition of HBSS+HEPES solution containing morphine (1 µM) or M6G (1 µM) in the presence and absence of inhibitor for transporter (1mM probenecid and tetraethylammonium) to apical insert for apical to basal transport or to basal insert for basal to apical transport. Samples were collected from basal (for apical to basal transport) or apical (for basal to apical transport) insert and analyzed by LC/MS/MS. Concentrations were used to determine apparent permeability (P_app_) by using the following equation:

$P_{app}= \frac{{\Delta Q}/{\Delta t}}{C_{0}*A}$ (4)

where ∆Q⁄∆t is the rate of morphine or M6G transport into basal or apical insert, A is the surface area of the insert and C_0_ is the initial concentration of morphine or M6G. Prediction of renal clearance from 2D Transwell^TM^ study was conducted as described previously using a 35-compartment mechanistic kidney model based on the MATLAB and Simulink platforms ^1^. Since no active secretion was observed in PTEC monolayer culture in 2D Transwell^TM^, active secretion clearance was considered as zero for predictions.

### Metabolism and hepatic transport in human hepatocyte MPS and hepatic uptake study of morphine in plated human hepatocytes

Cytochrome P450 enzyme and hepatic transporter activity qualified cryopreserved human hepatocytes (HUM190131) were purchased from Lonza (Walkersville, MD). Human hepatocyte MPS was prepared as described previously with some modification ^2^. 0.2 to 0.3 mL of human hepatocytes suspension a density of 4 × 10^6^ hepatocytes/mL were seeded to MPS chamber pre-coated with type I collagen solution (120 µg/mL, Ibidi USA, Inc. Madison, Wisconsin) in 0.1% acetic acid. The day after seeding cells, the hepatocytes were switched to ice-cold Matrigel containing (Corning, Oneonta, NY, final concentration 0.4 mg/mL) hepatocyte maintenance media. On the day after Matrigel overlay, perfusion was initiated at a rate of 1.0 µL/min with hepatocyte maintenance media. The day after starting perfusion, metabolism and transport study in MPS was carried out by starting perfusion of hepatocyte maintenance media containing morphine (1 µM) in the presence or absence of competitive inhibitors for organic cation transporters (tetraethylammonium (1mM)) at 1 µL/min. Effluents from MPS were collected every two hours, and drug concentrations in the samples were determined by LC/MS/MS. Uptake study of morphine by plated human hepatocytes was conducted as described previously ^3^. Briefly, the cryopreserved human hepatocytes (lot HUM190131) were thawed and seeded (5.0 × 10^5^ cells/well) into 24-well plated coated with collagen Ⅰ. At 6 hours post seeding, uptake study was conducted by replacing the culture media with pre-warmed HBSS buffer containing morphine (1 µM) in the presence or absence of OCTs inhibitors (1 mM tetraethylammonium). The reactions were terminated at 15 min by washing the cells three times with ice-cold HBSS buffer and cells were lysed with methanol. The samples were analyzed by LC-MS/MS.


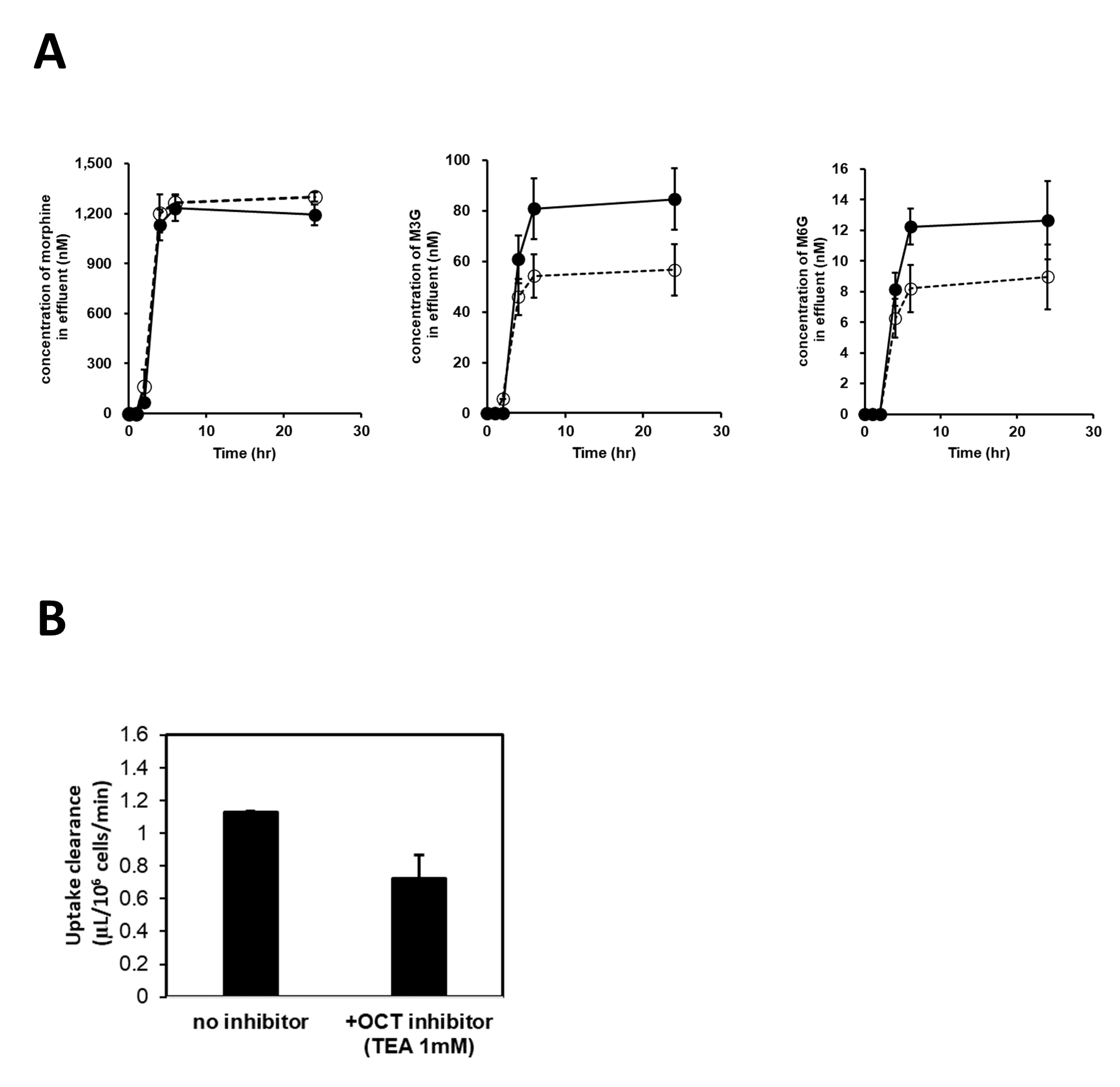


**Supplemental Figure 1.** Metabolism and transport of morphine using liver MPS

(A) Concentration-time profiles of Morphine (left), M3G (middle) and M6G (right) in the effluents of human liver MPS. Morphine was administered to liver MPS in the presence (dashed line) and absence (solid line) of OCTs inhibitors and effluents were collected and concentrations were measured. Data represents mean ± SD. (B) Effect of OCTs inhibitors on hepatic uptake of morphine by human plated hepatocytes. Morphine (1 µM) was incubated in human plated hepatocytes for 15 minutes and uptake of morphine was measured (mean ± SD).

### Supplemental Table 1. Donor information on kidney tissues used for isolation of proximal tubule epithelial cells.

Abbreviations: RCC, kidney cell carcinoma.

Kidney tissue was obtained in accordance with a protocol approved by the University of Washington Human Subjects Institutional Review Board (IRB STUDY00001297).

| **Donor (ID)** | **Age** | **Sex** | **Ethnicity** | **Pre-existing Conditions** | **Final Pathology** |
| --- | --- | --- | --- | --- | --- |
| 1  (Bio71) | 55 | Male | White | Not available | Clear cell RCC |
| 2  (Bio102) | 59 | Male | Native American | Not available | Urothelial Cell Carcinoma |
| 3  (Bio92) | 64 | Male | White | End stage kidney disease, Coronary Artery Disease | RCC |
| 4  (Bio38) | 74 | Male | Black or African American | Chronic Kidney Disease, Anemia, Gout, Hypertension, Diabetes | Chromophobe RCC |
| 5  (Bio144) | 69 | Male | Asian | Hypertension, Vertigo, | Chromophobe RCC |
| 6  (Bio145) | 64 | Male | White | Not available | Papillary Kidney Cell Carcinoma |
| 7  (Bio136) | 67 | Male | White | Chronic lymphocytic leukemia, Depression, Arthritis, Disorder of muscle, ligament, and fascia | Clear cell RCC |
| 8  (Bio139) | 57 | Male | Asian | Hypertension, Lipidemia, Type 1 diabetes | Clear cell RCC |

### Supplemental Table 2. Transport of morphine and M6G by VPT-MPS

Kinetic parameters used for calculation of CL_app, passive diffusion_, MPS and CL_app, active secretion_ from Fig 2. Data represents mean ± SD

|  | Morphine | | M6G | |
| --- | --- | --- | --- | --- |
|  | Without inhibitor | With inhibitor | Without inhibitor | With inhibitor |
| Efflux rate into lumen  (pmol/min/MPS) | 0.283 ± 0.116 | 0.0634 ± 0.0500 | 0.110 ± 0.043 | 0.0191 ± 0.0192 |
| Concentration in endothelial cell  (pmol/mL) | 1870 ± 39 | 1794 ± 61 | 640 ± 58 | 639 ± 34 |

### Supplemental Table 3. TEER and Papp values for the bidirectional transport of Morphine, M6G and [^3^H]PAH across PTECs and MDCK monolayers after 1.5 hour incubation in conventional 2D Transwell^TM^.

Data represents mean ± SD from three different donors

|  | | | Donor2 | Donor3 | Donor4 | Donor5 | Donor6 | Donor7 | Donor8 | MDCK |
| --- | --- | --- | --- | --- | --- | --- | --- | --- | --- | --- |
| TEER (ohm∙cm^2^) | | | 31.0± 10.3 | 28.1± 9.4 | 136± 45.5 | 119± 40 | 44.5± 14.8 | 431± 144 | 858± 286 | 723± 51 |
| Morphine | Inhibitor (-) | Apical to Basal  (10^-6^ cm/s) | 62.3 | 36.5 ± 1.1 | 31.9 | 29.6 ±2.3 | 57.9 | - | - | 5.49 ± 4.56 |
|  | Inhibitor (-) | Basal to Apical  (10^-6^ cm/s) | 59.6 | 23.0 ± 3.0 | 14.3 | 30.1 ± 6.4 | 48.5 | - | - | 4.19 ± 1.54 |
|  | Inhibitor (+) | Apical to Basal  (10^-6^ cm/s) | 65.8 | 39.2 ± 3.6 | - | 28.7 ± 2.7 | 58.2 | - | - | - |
|  | Inhibitor (+) | Basal to Apical  (10^-6^ cm/s) | 54,6 | 28.3 ± 3.8 | - | 30.9 ± 13.7 | 49.7 | - | - | - |
| M6G | Inhibitor (-) | Apical to Basal  (10^-6^ cm/s) | 58.0 | 40.1 ± 7.5 | 22.5 | 28.6 ± 1.6 | 55.6 | - | - | 3.86 |
|  | Inhibitor (-) | Basal to Apical  (10^-6^ cm/s) | 54.6 | 24.9 ± 14.2 | 12.2 | 28.6 ± 2.1 | 42.2 | - | - | 1.53 ± 0.17 |
|  | Inhibitor (+) | Apical to Basal  (10^-6^ cm/s) | 57.7 | 37.5 ± 4.7 | - | 25.0 ± 1.0 | 50.1 | - | - | - |
|  | Inhibitor (+) | Basal to Apical  (10^-6^ cm/s) | 60.1 | 27.1 ± 8.6 | - | 26.8 ± 4.8 | 52.6 | - | - | - |
| [^3^H] PAH | Inhibitor (-) | Apical to Basal (10^-6^ cm/s) | 72.4 | 31.7 | - | - | 71.7 | - | - | - |
|  | Inhibitor (-) | Basal to Apical (10^-6^ cm/s) | 69.6 | 23.0 | - | - | 67.3 | - | - | - |
|  | Inhibitor (+) | Apical to Basal (10^-6^ cm/s) | 75.6 | 32.9 | - | - | 67.1 | - | - | - |
|  | Inhibitor (+) | Basal to Apical (10^-6^ cm/s) | 76.3 | 30.6 | - | - | 68.2 | - | - | - |

### Supplemental Table 4. Prediction of renal clearance of morphine and M6G from PTECs permeability study data

Data represents mean ± SD from three different donors

|  | **Morphine** | | | | | **M6G** | | | | | |
| --- | --- | --- | --- | --- | --- | --- | --- | --- | --- | --- | --- |
|  | Donor2 | Donor3 | Donor4 | Donor5 | Donor6 | Donor2 | Donor3 | Donor4 | Donor5 | Donor6 |  |
| **Papp**  **(10^-6^ cm/s)** | 62.3 | 36.5 | 31.9 | 29.6 | 57.9 | 58.0 | 40.1 | 22.5 | 28.6 | 40.1 |  |
| **CL_int,_ _secretion_ (**µ**L/hr)** | 0 | 0 | - | 0 | 0 | 0 | 0 | - | 0 | 0 |  |
| **CL_r,predicted_ (L/hr)** | 1.84 | 2.65 | 2.88 | 3.01 | 1.94 | 3.44 | 3.98 | 4.72 | 4.43 | 3.98 |  |
| **CL_r,observed_ (L/hr)** | 6.8-9.62 ^4-6^ | | | | | 9.20-14.3 ^5,6^ | | | | | |

**References**

1 Huang, W. & Isoherranen, N. Development of a Dynamic Physiologically Based Mechanistic Kidney Model to Predict Renal Clearance. *CPT Pharmacometrics Syst Pharmacol* **7**, 593-602, doi:10.1002/psp4.12321 (2018).

2 Chang, S. Y. *et al.* Characterization of rat or human hepatocytes cultured in microphysiological systems (MPS) to identify hepatotoxicity. *Toxicol In Vitro* **40**, 170-183, doi:10.1016/j.tiv.2017.01.007 (2017).

3 Bi, Y. A. *et al.* Reliable Rate Measurements for Active and Passive Hepatic Uptake Using Plated Human Hepatocytes. *AAPS J* **19**, 787-796, doi:10.1208/s12248-017-0051-2 (2017).

4 Crews, K. R. *et al.* Lack of effect of ondansetron on the pharmacokinetics and analgesic effects of morphine and metabolites after single-dose morphine administration in healthy volunteers. *Br J Clin Pharmacol* **51**, 309-316, doi:10.1046/j.1365-2125.2001.01369.x (2001).

5 Hasselstrom, J. & Sawe, J. Morphine pharmacokinetics and metabolism in humans. Enterohepatic cycling and relative contribution of metabolites to active opioid concentrations. *Clin Pharmacokinet* **24**, 344-354, doi:10.2165/00003088-199324040-00007 (1993).

6 Somogyi, A. A. *et al.* Plasma concentrations and renal clearance of morphine, morphine-3-glucuronide and morphine-6-glucuronide in cancer patients receiving morphine. *Clin Pharmacokinet* **24**, 413-420, doi:10.2165/00003088-199324050-00005 (1993).
